# Acute stress enhances the glutamatergic transmission onto basoamygdala neurons embedded in distinct microcircuits

**DOI:** 10.1186/s13041-016-0283-6

**Published:** 2017-01-09

**Authors:** Chen Song, Wen-Hua Zhang, Xue-Hui Wang, Jun-Yu Zhang, Xiao-Li Tian, Xiao-Ping Yin, Bing-Xing Pan

**Affiliations:** 1 Laboratory of Fear and Anxiety Disorders, Institute of Life Science, 330031 Nanchang, China; 2 College of Life Science, 330031 Nanchang, China; 3 Department of Neurology, the 2nd affiliated Hospital, Nanchang University, 330031 Nanchang, China; 4 Jiangxi Provincial Collaborative Innovation Center for Cardiovascular, Digestive and Neuropsychiatric diseases, 330031 Nanchang, China

**Keywords:** Amygdala, Acute stress, Intrinsic excitability, Medial prefrontal cortex, Spontaneous postsynaptic current

## Abstract

Amygdala activation is known to be critical for the processing of stressful events in brain. Recent studies have shown that the projection neurons (PNs) in amygdala, although architecturally intermingled, are integrated into distinct microcircuits and thus play divergent roles in amygdala-related behaviors. It remains unknown how stress regulates the individual amygdala PNs embedded in distinct microcircuits. Here, by using retrograde tracing and electrophysiological recording in in vitro slices, we explored the modulation of acute immobilization stress (AIS) on the basoamygdala (BA) PNs projecting either to medial prefrontal cortex (mPFC) or elsewhere, which we designated as BA-mPFC and non-BA-mPFC PNs respectively. The results showed that in the control mice, both the excitatory and inhibitory postsynaptic currents (sEPSCs/sIPSCs) were comparable between these two subsets of BA PNs. The influences of AIS on sEPSCs and sIPSCs were overall similar between the two neuronal populations. It markedly increased the sEPSCs amplitude but left unaltered their frequency as well as the sIPSCs amplitude and frequency. Despite this, several differences emerged between the effects of AIS on the distribution of sEPSCs/sIPSCs frequency in these two groups of BA PNs. Similar changes were also observed in the sEPSCs/sIPSCs of the two PN populations from mice experiencing forced swimming stress. Their intrinsic excitability, on the other hand, was nearly unaltered following AIS. Our results thus suggest that acute stress recruit both BA-mPFC and non-BA-mPFC PNs mainly through enhancing the glutamatergic transmission they receive.

**Electronic supplementary material:**

The online version of this article (doi:10.1186/s13041-016-0283-6) contains supplementary material, which is available to authorized users.

## Background

Effectively coping with the stressful events in daily life is critical for the survival of organisms [1]. It has been known for decades that the stress coping system is evolutionally conservative in brain across species ranging from rodents to primates and to humans [2]. Amygdala is one of the kernel parts of this system and responsible for receiving and integrating different modes of information from sensory cortex and thalamus and passing them down to the executive nuclei in the hypothalamus or brainstem to elicit a spectrum of stress responses [3], including increased startle reactivity, heightened autonomic tone and activation of neuroendocrine axes [4, 5]. As such, amygdala activation has been generally regarded as an important neuronal correlate for stress processing inside the brain [6–9].

As commonly known, amygdala is a complex composed of more than ten sub-nuclei [10]. Among them, the basal part of amygdala (BA) acts to bridge the information flow from the lateral amygdala, the main reception of sensory information entering amygdala to the central amygdala, the main exit of information processed inside amygdala [11]. It also accounts for the intercommunication between amygdala and many other regions including prefrontal cortex, hippocampus and ventral striatum [10]. One prominent architectural feature which distinguishes BA from its neighboring regions is that the projection neurons (PNs) in this region, unlike those in cerebral cortex or hippocampus exhibiting clear laminar and columnar organization, are extensively intermingled [12]. Despite this, increasing evidence in recent years has demonstrated that the intermingled BA neurons are integrated into distinct functional circuits and thus have divergent or even opposing roles in the processing of emotionally salient events in amygdala [13–16]. For example, optogenetic activation of the BA terminals projecting to nucleus accumbens (NAc) drives positive reinforcement while BA projections to the central amygdala are related with negative valence [14].

Given the critical role of amygdala activation in brain processing of stress [9], it remains unknown how stress exposure affects the BA PNs integrated into distinct functional circuits. It has been recently reported that the BA PNs projecting to mPFC nearly non-overlap anatomically with those projecting to elsewhere such as hippocampus [17], we here attempted to explore the modulation of AIS on distinct BA PNs based on whether they make synaptic connections with mPFC. The results showed that acute stress regulated both BA-mPFC and non-BA-mPFC PNs mainly through enhancing the glutamatergic transmission they received. By contrast, it did not affect the intrinsic excitability of both subsets of BA PNs.

## Methods

### Animals

Female 129S1/SvlmJ mice were subject to acute immobilization stress at age of 8–10 weeks. The mice were housed in groups of 3–5 with *ad libitum* access to food and water in a temperature and humidity controlled facility with a 12/12 h light/dark cycle. All experiments were performed under the guidance of National Institutes of Health and with the approval of the Institutional Animal Care and Use Committee of Nanchang University.

### Stereotaxic surgery and injections of retrobeads

As previously descried [18], the stereotaxic injections of retrobeads were performed 10 days prior to acute stress on mice under general anesthesia of 2% pentobarbital sodium (4.5 ml/kg) by using stereotaxic instrument (Stoelting Co.). To label BA-mPFC PNs, the red retrobeads (Red Retrobeads^TM^ IX, Lumafluor Inc.) were bilaterally injected into the mPFC (0.5 μl per side) at stereotaxic coordinates (1.7 mm rostral to bregma, ± 0.4 mm lateral to midline, and 2.6 mm ventral to bregma). Injections were performed using glass micropipettes with their tip diameters of about 10–20 μm (pulled with the Narishige PC-10 puller). Our preliminary experiments have shown that injection with the micropipette results in less staining in the injection tracts compared to that by 1 μl Hamilton Syringe. After injection, the pipette was left in the injection site for an additional 10 min before being pulling out slowly. The mice were moved to their home cages after full recovery from anesthesia.

### Acute Immobilization Stress (AIS)

To subject the mice to AIS, we placed them in a plastic restraint cylinder fitted closely to its body size and drilled with some holes to allow free breathing at around 2 pm for 2 h. The mice assigned in the control group were transferred in their home cages to the experimental room with gentle handling for 24 min and sacrificed for eletrophysiological experiment about 2 h later.

### Forced Swimming Stress (FSS)

Mice were forced to swim for 10 min in a glass breaker containing water at 25 °C and having an internal diameter of 15 cm. The water with a depth of 12 cm permitted the mice to reach the bottom with their tails only. After completion of the swimming procedure, mice were carefully dried with a towel and put back to their home cage for about 1/2 h before the electrophysiological experiment.

### Electrophysiology

The experiment was performed as we described previously [19]. Briefly, the mice were anesthetized with ether and decapitated upon the cessation of stress. Brains were removed from the skull quickly and chilled in ice-cold artificial cerebrospinal fluid (ACSF) containing (in mM) 124 NaCl, 2.5 KCl, 2 MgSO_4_, 2.5 CaCl_2_, 1.25 NaH_2_PO_4_, 22 NaHCO_3_, and 10 glucose, bubbled with 95% O_2_ and 5% CO_2_. Coronal brain slices of 300 μm thickness containing the amygdala were cut using the VT1000S Vibratome (Leica Microsystems). The slices were recovered in ACSF for 30 min at 34 °C. Later on, the slices were removed to the incubator at room temperature for at least 1 h before the experiment commenced.

During the experiment, slices were transferred to the recording chamber and continuously perfused with the ACSF. The filamented borosilicate glass capillary tubes (inner diameter, 0.89 μm) were pulled using a horizontal pipette puller (P-97; Sutter Instrument) to prepare recording electrodes. The experimenter for the patch-clamp recordings and analyses were blind to the group into which the mice were assigned. For recordings of sEPSCs and sIPSCs in BA PNs, the patch electrodes (2–3 MΩ resistance) were filled with Cs^+^-based pipette solution containing (in mM) 130 Cs-methanesulfonate, 5 NaCl, 1 MgCl_2_, 10 HEPES, 0.2 EGTA, 2 MgATP, and 0.1 NaGTP. The pH was adjusted to 7.3 with CsOH and osmolarity to 285 mOsm with sucrose. 10 μM bicuculline was added to block A type of GABA receptor currents during recording of sEPSCs and 20 μM CNQX and 20 μM APV were used to block ionotropic glutamate receptor currents during recording of sIPSCs. In experiments where action potentials were evoked, Cs-methanesulfonate was replaced by equal concentrations of K-gluconate. All recordings were performed at room temperature using MultiClamp 700B amplifier (Molecular Devices). The membrane potentials were held at −55 and 0 mV for recording of sEPSCs and sIPSCs respectively in a voltage-clamp mode. To evoke action potentials in the PNs, cells were recorded at current clamp mode and the depolarizing current pulses were delivered. A junction potential of about 12 mV was not corrected. Series resistance (Rs) was in the range of 10–20 MΩ and monitored throughout the experiments. If Rs changed more than 20% during recording, the data were not included in analysis. Offline data analysis was performed using MiniAnalysis and Clampfit 9 program (Molecular Devices).

### Statistics

Data are expressed as mean ± SEM. Appropriate statistical approaches including the unpaired *t* tests, *two-way* and *multi-way* ANOVA were used followed by *post hoc* comparison with Bonferroni-corrected *t* test. The distributions of current amplitude and frequency were examined by Kolmogorov-Smirnov test. The *p* value of less than 0.05 was considered statistically different. All statistical analyses were conducted using Prism version 6.0 (GraphPad Software).

## Results

### AIS markedly enhances the glutamatergic transmission onto both BA-mPFC and non-BA-mPFC PNs

Before examining the modulation of AIS on BA-mPFC and non-BA-mPFC PNs, we first tested its potential influence on BA PNs as a whole. Relative to those from the control mice, the BA PNs from AIS mice had sEPSCs with higher amplitude (*p* = 0.023, control, *n* = 10 cells/3 mice; AIS, *n* = 10 cells/3 mice, unpaired *t* test, Additional file 1: Figure S1a-b). By contrast, no between-group difference was observed in their frequency (*p* = 0.845, unpaired *t* test, Additional file 1: Figure S1b). Consistently, the distribution of sEPSC amplitude (*p* = 0.002; Additional file 1: Figure S1c) but not frequency (*p* = 0.781; Additional file 1: Figure S1d) exhibited marked differences between BA PNs from control and AIS mice. Thus, it appears that AIS augments the glutamatergic transmission onto BA PNs mainly through a postsynaptic mechanism.

**Fig. 1:**
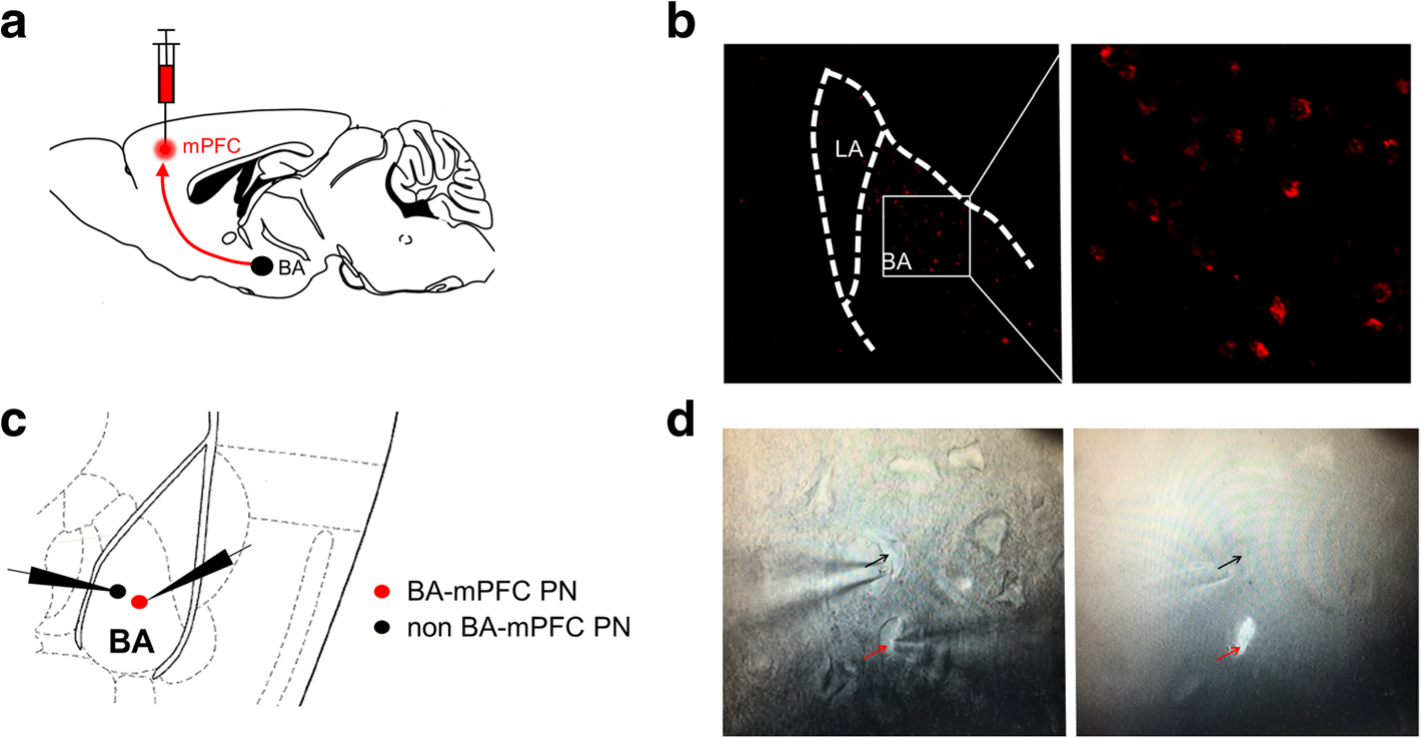
Simultaneous whole-cell recording of BA-mPFC and non-BA-mPFC neurons. **a** Schematic diagrams showing the injection of red retrobeads into the medial prefrontal cortex (mPFC) to label the basal amygdala (BA) neurons projecting to the mPFC. **b** The fluorescent image showing the staining of BA-mPFC PNs by the red retrobeads injected in the mPFC. **c** Schematic diagrams showing simultaneous whole-cell recordings of the BA-mPFC and non-BA-mPFC PNs. **d** Images taken under the bright field (*left*) or fluorescent microscope (*right*) showing paired recording of one BA-mPFC PN (*red arrow*) and one of its proximal non-BA-mPFC PNs (*black arrow*)

**Fig. 2:**
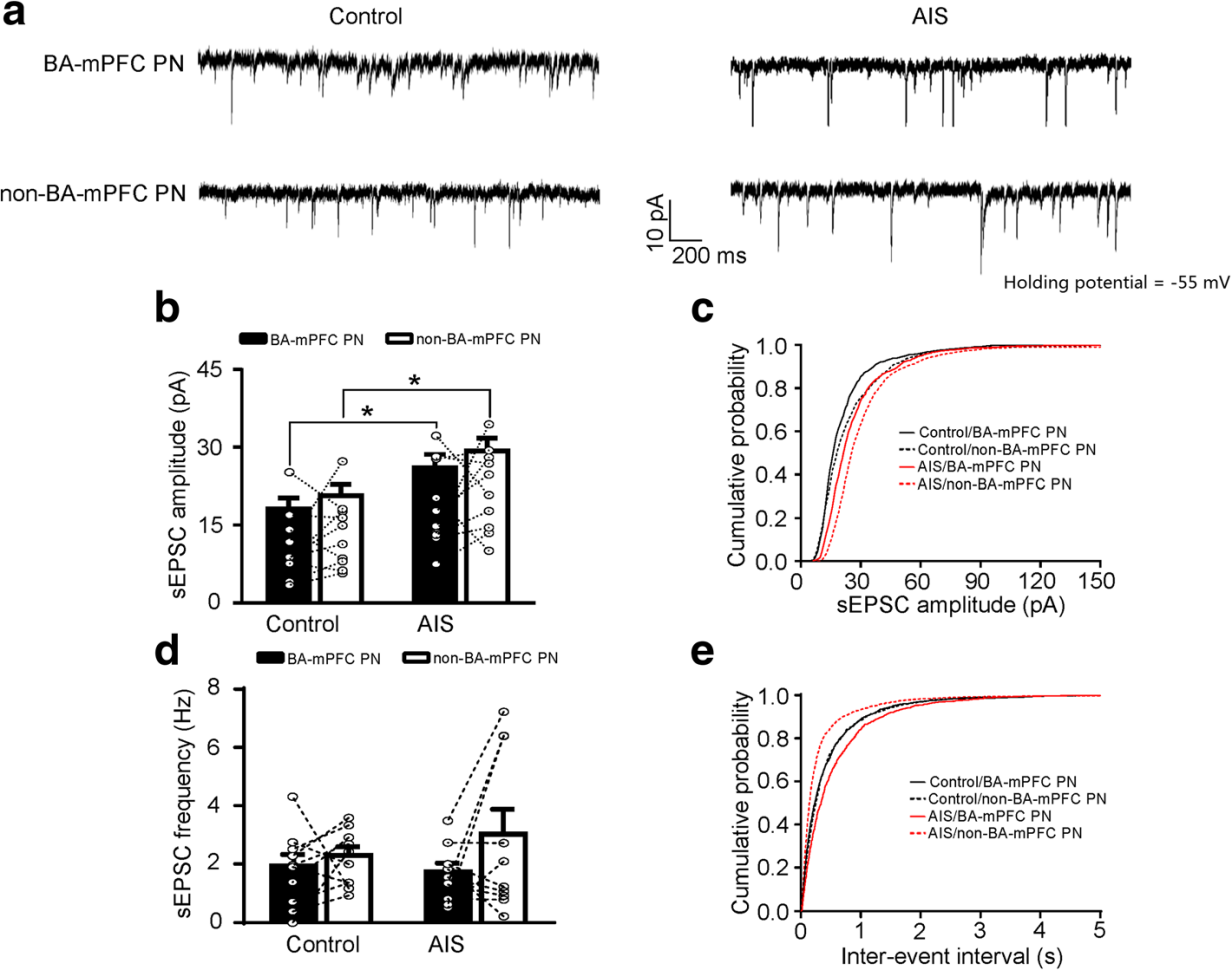
AIS significantly augments the glutamatergic transmission onto both BA-mPFC and non-BA-mPFC neurons. **a** Representative traces showing sEPSCs recorded from the BA-mPFC PNs and their neighboring non-BA-mPFC PNs in the control and AIS mice. **b** Summary data showing the sEPSCs amplitude of the pair-recorded BA-mPFC and non-BA-mPFC PNs (*in circle*) from the control and AIS mice. Their mean values were shown in column. **c** Cumulative distribution of the sEPSC amplitude in the two BA neuronal subsets. **d** Summary data of the sEPSC frequency in both BA neuronal subsets. **e** Cumulative distribution of the sEPSC frequency in both BA neuronal subsets. *p < 0.05, **p < 0.01

We next investigated the specific influence of AIS on the sEPSCs in BA-mPFC and non-BA-mPFC PNs. These two subsets of BA PNs could be readily differentiated based on the presence or absence of the red retrobeads in their soma (Fig. 1a-b). Simultaneous whole-cell recordings were made from one randomly selected BA-mPFC PN and one of its neighboring non-BA-mPFC PN in the same slices (Fig. 1c-d). *Two-way* ANOVA (neuron subset × AIS) revealed AIS but not neuronal subset had significant main effect on the sEPSC amplitude (AIS: F_(1, 36)_ = 12.42, *p* = 0.001; neuronal subset: F_(1, 36)_ = 1.515, *p* = 0.226; control, *n* = 11 pairs/4 mice; AIS, *n* = 10 pairs/3 mice; Fig. 2a-b). *Post-hoc* analysis revealed that AIS greatly increased the sEPSC amplitude in both neuronal subsets (BA-mPFC PN: *p* = 0.029; non-BA-mPFC PN: *p* = 0.018, Fig. 2b). Moreover, AIS considerably right-shifted the distribution of sEPSC amplitude in these neurons (BA-mPFC PNs, *p* < 0.001; non-BA-mPFC PNs, *p* < 0.001, Fig. 2c). By contrast, neither AIS nor neuronal subset had significant influence on the mean value of sEPSCs frequency (AIS: F_(1, 36)_ = 0.266, *p* = 0.609; neuronal subset: F_(1, 36)_ = 2.545, *p* = 0.119; Fig. 2d). Despite this, AIS appeared to have significant but contrasting influences on their distribution in these two neuronal subsets. While causing a right-shift of the inter-event interval in BA-mPFC PNs (*p* < 0.001 vs. control, Fig. 2e), AIS left-shifted that in non-BA-mPFC PNs (*p* < 0.001; Fig. 2e). Thus, upon AIS, the sEPSCs frequency tended to be increased in the non-BA-mPFC PNs but decreased in their proximal BA-mPFC PNs.

### AIS has minor influence on the GABAergic transmission to both BA-mPFC and non-BA-mPFC PNs

We next explored the possible influence of AIS on the GABAergic transmission onto the BA PNs. Unlike having marked impact on the glutamatergic transmission to the BA PNs as a whole, AIS only had minor influence on their sIPSCs. It failed to significantly alter both sIPSC amplitude (*p* = 0.410, control, *n* = 9 cells/3 mice; AIS, *n* = 9 cells/3 mice, Additional file 2: Figure S2a-b) and frequency (*p* = 0.708, Additional file 2: Figure S2b) although a marked right-shift of the distribution of sIPSC amplitude (*p* < 0.001, Additional file 2: Figure S2c) but not frequency (*p* = 0.126, Additional file 2: Figure S2d) was observed following AIS.

**Fig. 3:**
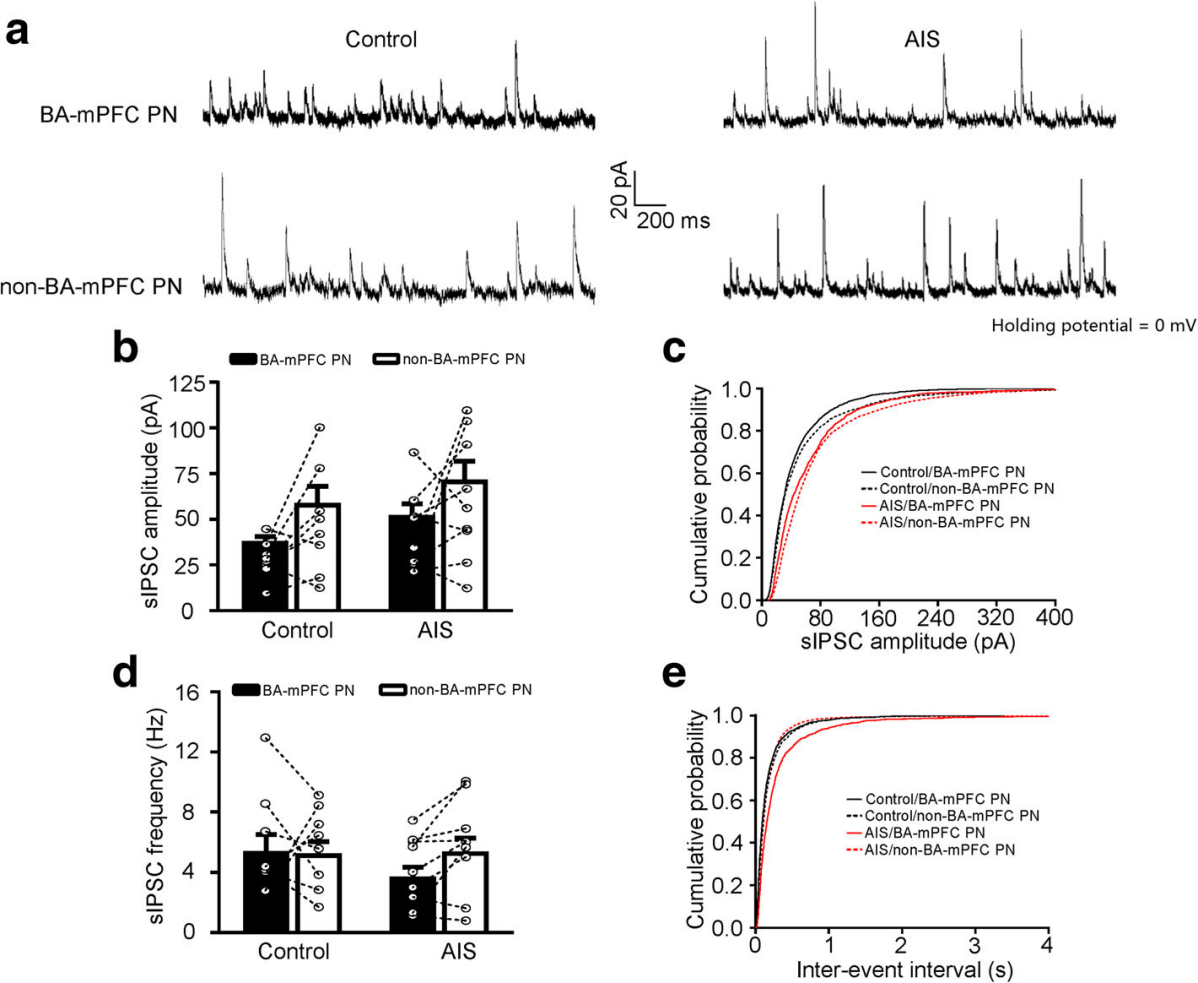
AIS slightly affects the GABAergic transmission onto both BA-mPFC and non-BA-mPFC neurons. **a** Representative traces showing sIPSCs recorded from the BA-mPFC PNs and their proximal non-BA-mPFC PNs in control and AIS mice. **b** Summary data showing the sIPSCs amplitude of the pair-recorded BA-mPFC and non-BA-mPFC PNs (*in circle*) from the control and AIS mice. Their mean values were shown in column. **c** Cumulative distribution of the sIPSC amplitude in both BA neuronal subsets. **d** Summary data of sIPSC frequency in both BA neuron subsets. **e** Cumulative distribution of the sIPSC frequency in both BA neuron subsets

Subsequent experiments using simultaneous recording of the two subsets of BA PNs revealed that neither AIS nor neuronal subset had significant main effect on the mean value of sIPSCs amplitude (AIS: F_(1, 30)_ = 2.286, *p* = 0.141; neuronal subset: F_(1, 30)_ = 5.160, *p* = 0.119; control, *n* = 8 pairs/3 mice; AIS, *n* = 9 pairs/3 mice; Fig. 3a-b). As with its influence on the distribution of sIPSCs amplitude in BA PNs as a whole, AIS also right-shifted the distribution in both PN subsets (BA-mPFC PNs, *p* < 0.001; non-BA-mPFC PNs, *p* < 0.001; Fig. 3c). On the other hand, neither AIS nor neuronal subset was found to significantly affect the sIPSC frequency (AIS: F _(1, 30)_ = 0.591, *p* = 0.448; neuronal subset: F_(1, 30)_ = 0.540, *p* = 0.468; control, *n* = 8 pairs/3 mice; AIS, *n* = 9 pairs/3 mice; Fig. 3d). Despite this, AIS right-shifted the distribution of sIPSCs frequency in BA-mPFC PNs (*p* < 0.001, Fig. 3e) but not their non-BA-mPFC counterparts (*p* = 0.112).

### FSS also enhances glutamatergic but not GABAergic transmission onto both BA-mPFC and non-BA-mPFC PNs

To test whether AIS-mediated preferential enhancement of glutamatergic transmission onto both PN populations also applies to mice experiencing other forms of stress, we repeated the above comparisons in mice subject to FSS for 10 min. Two-way ANOVA (neuron subset × FSS) revealed FSS but not neuronal subset had significant main effect on the sEPSCs amplitude (FSS: F_(1, 24)_ = 16.43, *p* < 0.001; neuronal subset: F_(1, 24)_ = 0.149, *p* = 0.703; control, *n* = 7 pairs/3 mice; FSS mice, *n* = 7 pairs/3 mice; Additional file 3: Figure S3a-b). *Post-hoc* analysis revealed that FSS greatly increased the sEPSCs amplitude in both neuronal subsets (BA-mPFC PN: *p* = 0.012; non-BA-mPFC PN: *p* = 0.016, Additional file 3: Figure S3b). Moreover, it considerably right-shifted the distribution of sEPSCs amplitude in BA-mPFC PNs (*p* = 0.019) but not in their non-BA-mPFC neighbors (*p* = 0.815, Additional file 3: Figure S3c). By contrast, neither FSS nor neuronal subset had significant influence on the mean value of sEPSCs frequency (FSS: F _(1, 24)_ = 0.726, *p* = 0.403; neuronal subset: F_(1, 24)_ = 2.015, *p* = 0.169; Additional file 3: Figure S3d). Similar to AIS, FSS had significant but contrasting influences on the distribution of sEPSCs frequency in these two neuronal subsets. It right-shifted the inter-event interval in BA-mPFC PNs (*p* < 0.001 vs. control), but left-shifted that in non-BA-mPFC PNs (*p* < 0.001; Additional file 3: Figure S3e).

The sIPSC amplitude, however, was unaltered in both PN populations by FSS (F_(1, 24)_ = 2.522, *p* = 0.125; control, *n* = 7 pairs/3 mice; FSS, *n* = 7 pairs/3 mice; Additional file 4: Figure S4a-b). However, it left-shifted the distribution of sIPSCs amplitude in the non-BA-mPFC PNs (*p* < 0.001) but not BA-mPFC PNs (*p* = 0.408; Additional file 4: Figure S4c). Similarly, despite the failure to affect the mean value of sIPSCs frequency (FSS: F_(1, 24)_ = 2.029, *p* = 0.167, Additional file 4: Figure S4d), it shifted the distribution of sIPSCs frequency in BA-mPFC PNs (*p* < 0.001, Additional file 4: Figure S4e) but not their non-BA-mPFC counterparts (*p* = 0.019).

### AIS unalters the intrinsic excitability of both BA-mPFC and non-BA-mPFC PNs

**Fig. 4:**
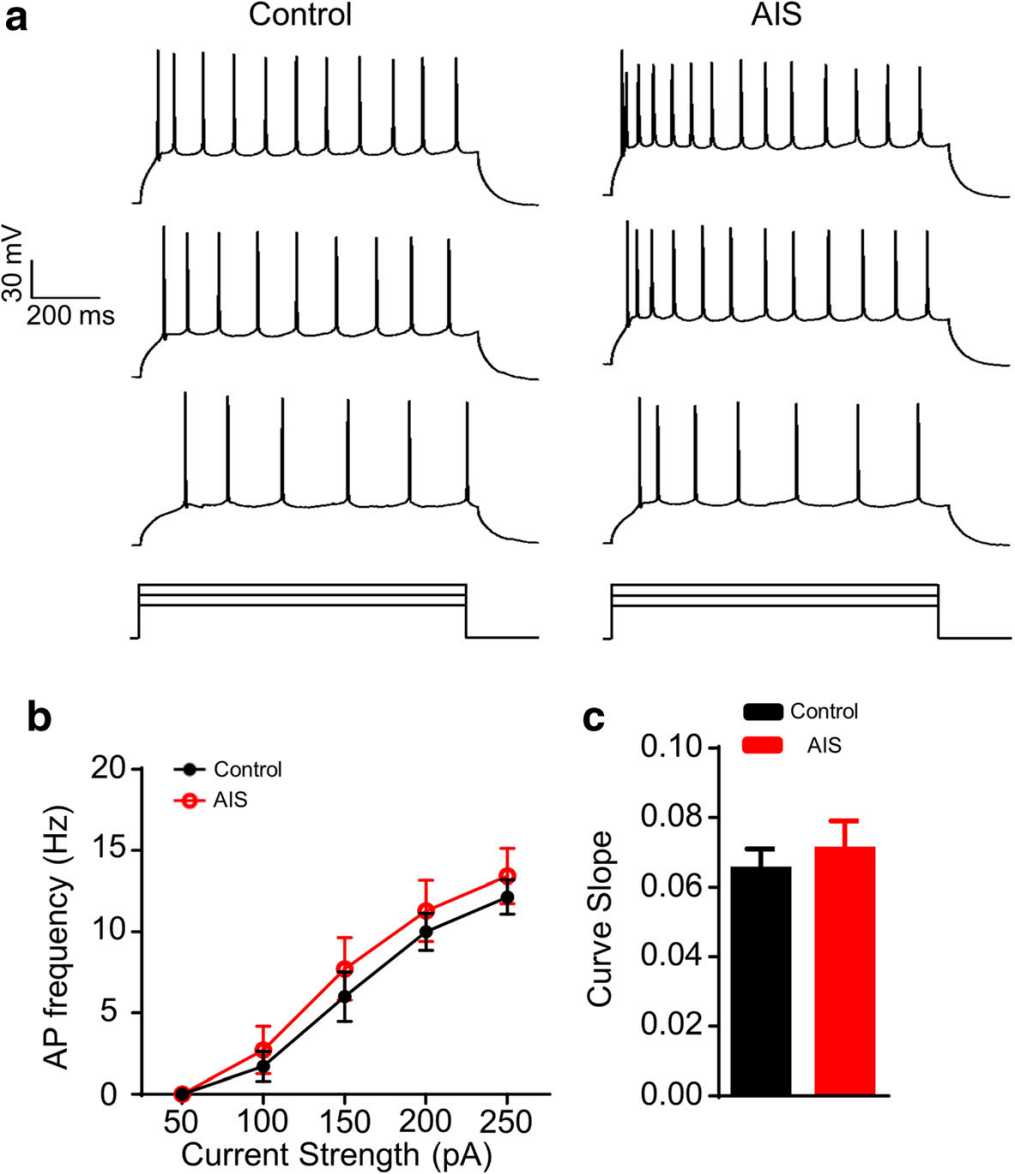
AIS unalters the intrinsic excitability of the BA PNs as a whole. **a** Representative traces showing the firing of BA PNs from the control (*left*) and AIS *(right)* mice upon the injection of depolarizing current pulses with varying strength from 150 (*bottom*), 200 (*middle*) to 250 pA (*top*). **b** Plot of the firing frequency of BA PNs from control and AIS mice as a function of the strength of the injected currents. **c** Comparison of the curve slope in (**b**)

To examine the potential regulation of the intrinsic excitability of BA PNs by AIS, we injected the recorded neurons with depolarizing current pulses with their strength being step increased at 50 pA. In line with the early findings that AIS had little influence on the neuronal excitability of BA PNs [19], we observed that AIS did not affect the number of action potentials in the whole BA PNs which were evoked at varying current strength (F_(1, 12)_ = 0.449, *p* = 0.515, control mice, *n* = 7 cells/3 mice, AIS mice, *n* = 7 cells/3 mice, Fig. 4a-b). Neither did it affect the slope of the fitted curve plotting the action potential number as a function of the current strength in individual neurons (*p* = 0.582; Fig. 4c).

**Fig. 5:**
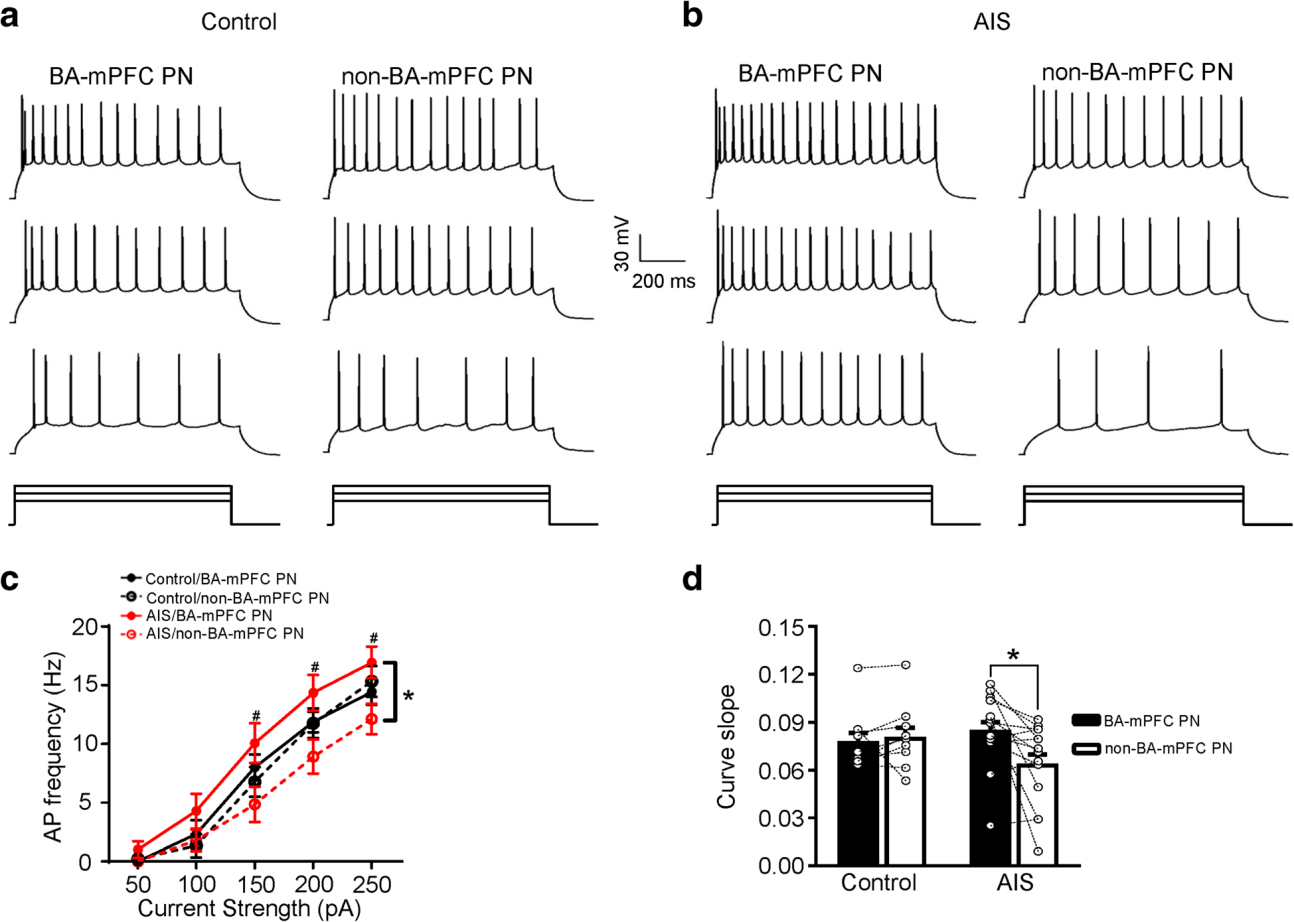
AIS has little influence on the intrinsic excitability of both BA-mPFC and non-BA-mPFC neurons. **a-b** Representative traces showing the firing of the pair-recorded BA-mPFC PNs (*left*) and their non-BA-mPFC counterparts (*right*) from control (**a**) and AIS (**b**) mice upon injection of current pulses with varying strength from 150 (*bottom*), 200 (*middle*) to 250 pA (*right*). **c** The plot of the firing frequency of distinct neuronal subsets as a function of the strength of the injected currents. **d** Comparisons of the curve slope in (**c**). *p < 0.05

Since the BA PNs tested in the above experiment represented a mixed group of BA-mPFC and non-BA-mPFC PNs, the absence of AIS modulation on the intrinsic excitability of the whole BA PNs may thus arise from two possibilities. First, it had little influence on the excitability of both neuronal subsets. Second, it enhanced the excitability of one subset but decreased that of the other. To differentiate these two possibilities, we next explored the specific modulation of AIS on the excitability of the two neuronal subsets. Multi-way RM ANOVA (neuron subset × AIS × current strength) revealed neuronal subset (BA-mPFC PNs, *n* = 23 cells/7 mice; non-BA-mPFC PNs, *n* = 23 cells/7 mice, *F* = 16.350, *p* < 0.001, Fig. 5a-c) but not AIS (control mice, *n* = 18 cells/3 mice; AIS mice, *n* = 28 cells/4 mice; *F* = 0.684, *p* = 0.409) had significant main effect on the number of the action potentials. Thus, AIS *per se* appeared not to significantly alter the excitability of these two BA neuronal subsets. The significant main effect of neuronal subtype was manifested by the different firing of these two PN subsets in the AIS rather than control mice. While both of them exhibited similar number of action potentials in the control mice (main effect of neuronal subtype, F_(1, 16)_ = 0.049, *p* = 0.827), the BA-mPFC PNs showed relatively stronger firing than their non-BA-mPFC counterparts in the AIS mice (main effect of neuronal subtype, F_(1, 26)_ = 5.951, *p* = 0.022; Fig. 5b). In accord with this, the curve slope of the BA-mPFC PNs was significantly higher than that of the non-BA-mPFC PNs in the AIS but not control mice (AIS: BA-mPFC PN: *p* = 0.016, *n* = 14 pairs/4 mice, paired *t* test; Fig. 5c).

## Discussion

In the present study, we aimed to explore the regulation of AIS on the excitatory and inhibitory transmission to the BA-mPFC and non-BA-mPFC PNs as well as their intrinsic excitability. The results showed that AIS had overall similar influences on these two PN populations. It markedly enhanced the sEPSCs in both of them but had little influence on the GABAergic transmission they received. Their intrinsic excitability, on the other hand, was nearly unaltered subsequent to AIS.

Amygdala is one of the key mediators of the influence of acute stress on emotion and cognition [20–22]. The enhanced glutamatergic transmission has been known to essentially account for the recruitment of amygdala by acute stress [23, 24]. As such, it was found to increase the release of glutamate and enhance the level of extracellular glutamate in BA [25], facilitating the delivery of AMPA receptor to the glutamatergic synapses with resultant augmentation of glutamatergic transmission [26]. In line with this, we also found that AIS markedly increased the sEPSC amplitude in BA PNs. More specifically, such an increase was evident in both BA-mPFC and non-BA-mPFC PNs, implying circuit-independent regulation of glutamatergic transmission to BA PNs by AIS. The increased sEPSC amplitude in both neuronal subsets suggests postsynaptic origin of the AIS-mediated enhancement of excitatory transmission in BA. Since acute stress was reported to exert similar alterations in other regions such as mPFC [27, 28] and hippocampus [26], it is reasonable to speculate that the postsynaptic effect by AIS may represent a common route through which it augments glutamatergic transmission in the limbic and cortical regions. On the other hand, we only observed very little changes in sEPSC frequency subsequent to AIS. This seems to be inconsistent with the early finding by Reznikov and his coworkers [25] that AIS readily increased the level of extracellular glutamate in BA. Such inconsistency may be partly due to the different experiment conditions used in our and Reznikov’s experiment. While we made the offline analysis of the glutamatergic transmission in the in vitro slice preparations, they conducted the real-time recording using HPLC inside the brain. It is thus likely that the increased glutamate release by acute stress, as observed by Reznikov et al., may diminish during the preparation of brain slices. Despite the failure to affect the sEPSC frequency as shown in the current study, AIS had subtle different effects on the distribution of sEPSC frequency in the BA-mPFC and non-BA-mPFC PNs. While the sEPSCs frequency in the BA-mPFC neurons tended to be increased upon AIS, that in the non-BA-mPFC neurons ended to be decreased. The synaptic mechanisms underlying this are virtually unclear and await further investigations.

We next observed that AIS had insignificant effect on the sIPSC amplitude in both BA PNs. However, its influence on the distribution of sIPSC amplitude was significant in these cells, reflecting increases in the fraction of sIPSCs with larger amplitudes. Actually, several earlier studies have found that acute stress enhanced the efflux of GABA in BA [29] and its neighboring central amygdala [30]. Thus, acute stress also recruits the inhibitory network in BA. Such recruitment, however, is somewhat at odds with the amygdala disinhibition following acute stress [20]. We speculate it may reflect a compensative response of the local inhibitory network, which may help to prevent excessive disinhibition in amygdala upon the emergence of acute stress and thus to ensure the appropriate stress response. Notably, the effects of AIS on both glutamatergic and GABAergic transmission in the two PN populations were readily mimicked by FSS, suggesting a possibility that different forms of stress may have similar influence on the synaptic transmission in BA.

Unlike the altered synaptic transmission by AIS onto the BA PNs, the intrinsic excitability of these PNs did not experience considerable changes subsequent to AIS. Similarly, a recent study reported that AIS also failed to affect the excitability of the PNs in lateral amygdala [31]. Given this, the recruitment of amygdala PNs by acute stress may be mainly achieved through enhancing the excitatory transmission they received rather than altering their intrinsic responsiveness. Notably, although AIS only had slight but statistically insignificant changes on the excitability of both neuronal subsets, the changes in the two populations appeared to occur along the opposite directions. While the excitability of the BA-mPFC PNs tended to be increased, that of the non-BA-mPFC PNs tended to be decreased. As a consequence, the BA-mPFC PNs fired more than their non-BA-mPFC counterparts in the AIS but not control mice. The functional significance of the relative enhancement of neuronal firing in BA-mPFC PNs is still in mystery but may help to facilitate the intercommunication between amygdala and prefrontal cortex [32, 33].

Although AIS failed to considerably alter the excitability of BA PNs, continuous exposure to the stressful events, on the other hand, was repetitively shown to increase the excitability of amygdala neurons [31, 34, 35]. Thus, the increased neuronal responsiveness subsequent to repeated stress exposure may reflect a persistent rather than transient influence of stress on BA neurons. Repeated stress has been known to cause a spectrum of enduring epigenetic changes, which may contribute to the structural and functional remodeling in amygdala neurons [36–38].

As stated in the introduction, the architecturally intermingled BA neurons are integrated into distinct microcircuits and thus play distinct roles in amygdala-related tasks [13, 14, 39]. Our current findings revealed that the AIS-mediated changes in BA-mPFC and non-BA-mPFC neurons were overall similar in terms of the excitatory and inhibitory transmission they received. Such nearly homogenous regulation by AIS may result in indistinguishable activation of the BA PNs embedded in distinct microcircuits. In support of this, we have recently observed that AIS results in similar level of increase in the c-fos expression in these two neuronal subsets which is indicative of neuronal activation (unpublished data). Interestingly, it was previously reported in human that acute stress augmented amygdala response to equally high level to both threat-related and positively-valenced stimuli [40]. The underlying neuronal mechanisms, however, remain elusive. The circuit-independent enhancement of glutamatergic transmission in BA may provide a neural basis for the recruitment of amygdala neurons by stress.

## Conclusions

Acute stress similarly enhances glutamatergic transmission onto the distinct BA PNs engaged in different circuits. Such modulation may underlie the region-wide activation of BA neurons by acute different circuits. Such modulation may underlie the region-wide activation of BA neurons by acutestress.

## Additional files

Additional file 1: Figure S1. AIS significantly augments the glutamatergic transmission onto BA PNs. **a** Representative traces showing the sEPSCs recorded from the BA PNs in control and AIS mice. **b** Summary data showing the sEPSCs amplitude (left) and frequency (right) of the BA PNs from control and AIS mice. **c**-**d** Cumulative distribution of the sEPSC amplitude (c) and frequency (d) in BA PNs. **p* < 0.05. (TIF 768 kb)
Additional file 2: Figure S2. AIS slightly affects the GABAergic transmission onto BA PNs. **a** Representative traces showing the sIPSCs recorded from the BA PNs in control and AIS mice. **b** Summary data showing the sIPSCs amplitude (left) and frequency (right) of the BA PNs from control and AIS mice. **c**-**d** Cumulative distribution of the sIPSC amplitude (c) and frequency (d) in BA PNs. (TIF 749 kb)
Additional file 3: Figure S3. FSS significantly augments the glutamatergic transmission onto both BA-mPFC and non-BA-mPFC neurons. **a** Representative traces showing sEPSCs recorded from the BA-mPFC PNs and their neighboring non-BA-mPFC PNs in the control and FSS mice. **b** Summary data showing the sEPSCs amplitude of the pair-recorded BA-mPFC and non-BA-mPFC PNs (in circle) from the control and AIS mice. Their mean values were shown in column. **c** Cumulative distribution of the sEPSC amplitude in the two BA neuronal subsets. **d** Summary data of the sEPSC frequency in both BA neuronal subsets. **e** Cumulative distribution of the sEPSC frequency in both BA neuronal subsets. **p* < 0.05. (TIF 1114 kb)
Additional file 4: Figure S4. FSS slightly affects the GABAergic transmission onto both BA-mPFC and non-BA-mPFC neurons. **a** Representative traces showing sIPSCs recorded from the BA-mPFC PNs and their proximal non-BA-mPFC PNs in control and FSS mice. **b** Summary data showing the sIPSCs amplitude of the pair-recorded BA-mPFC and non-BA-mPFC PNs (in circle) from the control and AIS mice. Their mean values were shown in column. **c** Cumulative distribution of the sIPSC amplitude in both BA neuronal subsets. **d** Summary data of sIPSC frequency in both BA neuron subsets. **e** Cumulative distribution of the sIPSC frequency in both BA neuron subsets. (TIF 1126 kb)

## Abbreviations

AIS: Acute immobilization stress
AMPA: 2-amino-3-(3-hydroxy-5-methyl-isoxazol-4-yl) propanoic acid
BA: Basoamygdala
FSS: Forced swimming stress
mPFC: Medial prefrontal cortex
NAc: Nucleus accumbens
PNs: Projection neurons
sEPSC: Spontaneous excitatory post-synaptic currents
sIPSC: Spontaneous inhibitory post-synaptic currents
