# Supplementary figures and images for: Acute stress enhances the glutamatergic transmission onto basoamygdala neurons embedded in distinct microcircuits
Source: Mol Brain. 2017 Jan 9;10:3. doi: 10.1186/s13041-016-0283-6 (PMC5223467; doi:10.1186/s13041-016-0283-6)

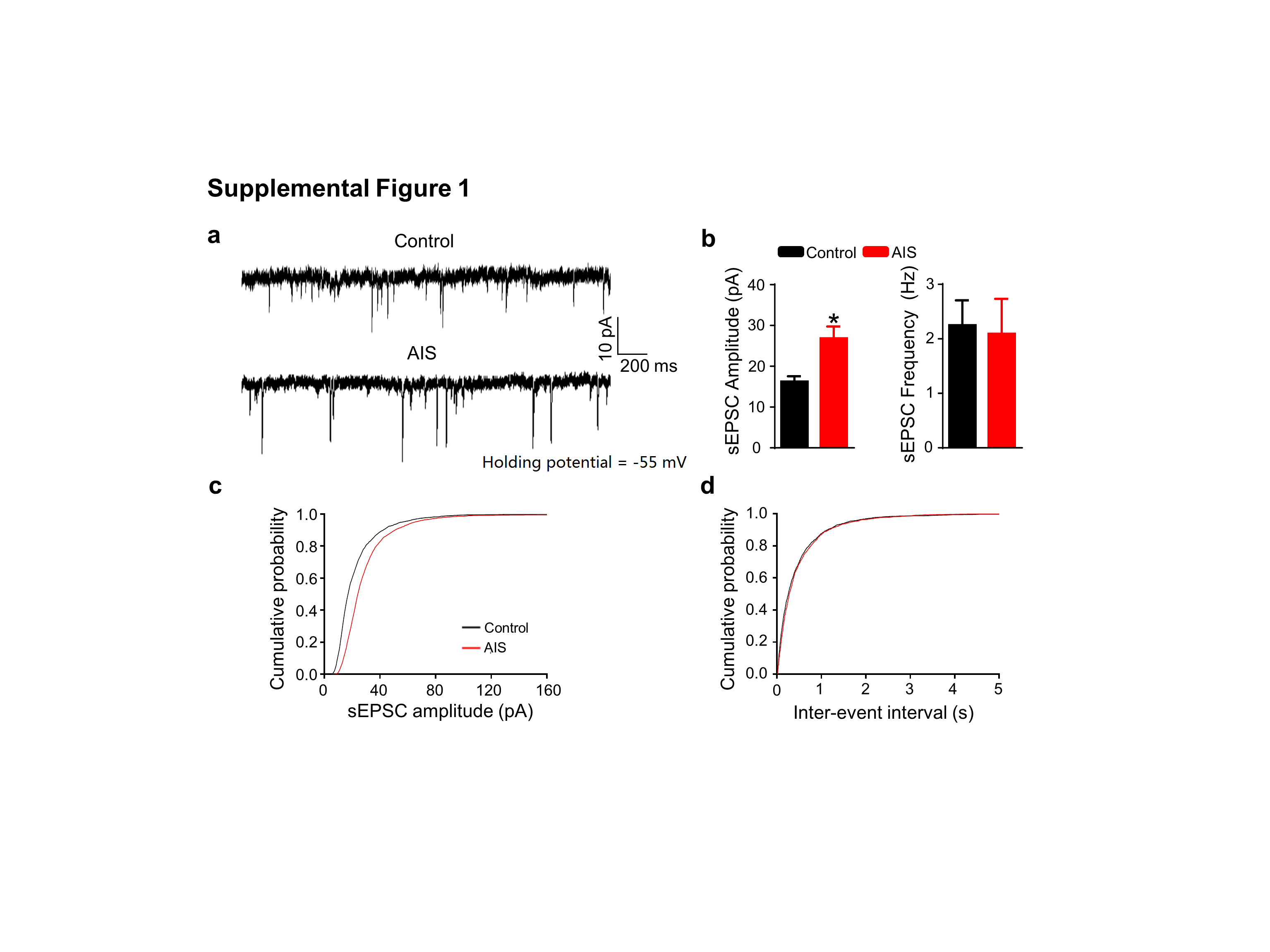

Supplement: Additional file 1: Figure S1. — AIS significantly augments the glutamatergic transmission onto BA PNs. a Representative traces showing the sEPSCs recorded from the BA PNs in control and AIS mice. b Summary data showing the sEPSCs amplitude (left) and frequency (right) of the BA PNs from control and AIS mice. c-d Cumulative distribution of the sEPSC amplitude (c) and frequency (d) in BA PNs. *p < 0.05. (TIF 768 kb) [file 13041_2016_283_MOESM1_ESM.tif]

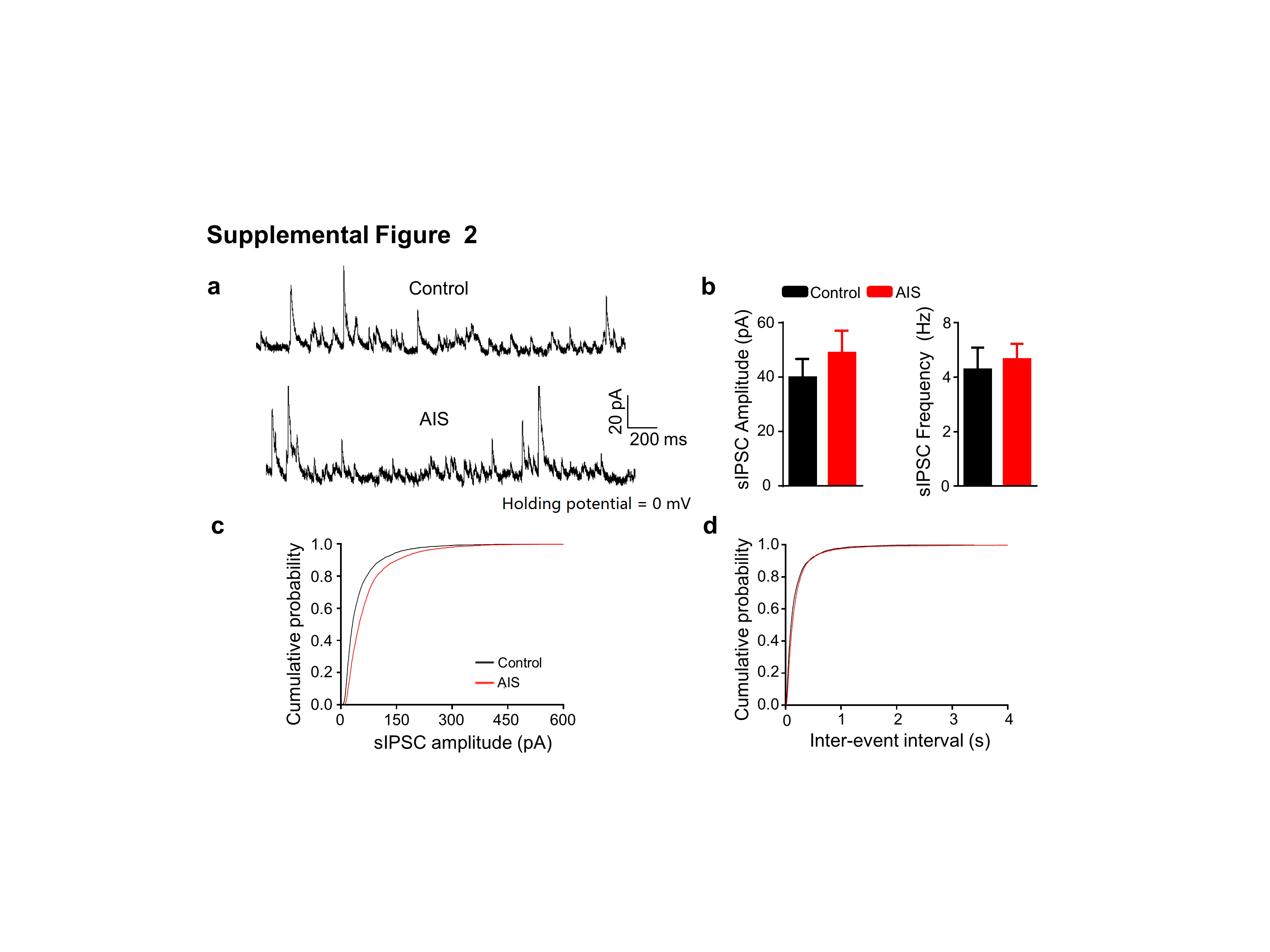

Supplement: Additional file 2: Figure S2. — AIS slightly affects the GABAergic transmission onto BA PNs. a Representative traces showing the sIPSCs recorded from the BA PNs in control and AIS mice. b Summary data showing the sIPSCs amplitude (left) and frequency (right) of the BA PNs from control and AIS mice. c-d Cumulative distribution of the sIPSC amplitude (c) and frequency (d) in BA PNs. (TIF 749 kb) [file 13041_2016_283_MOESM2_ESM.tif]

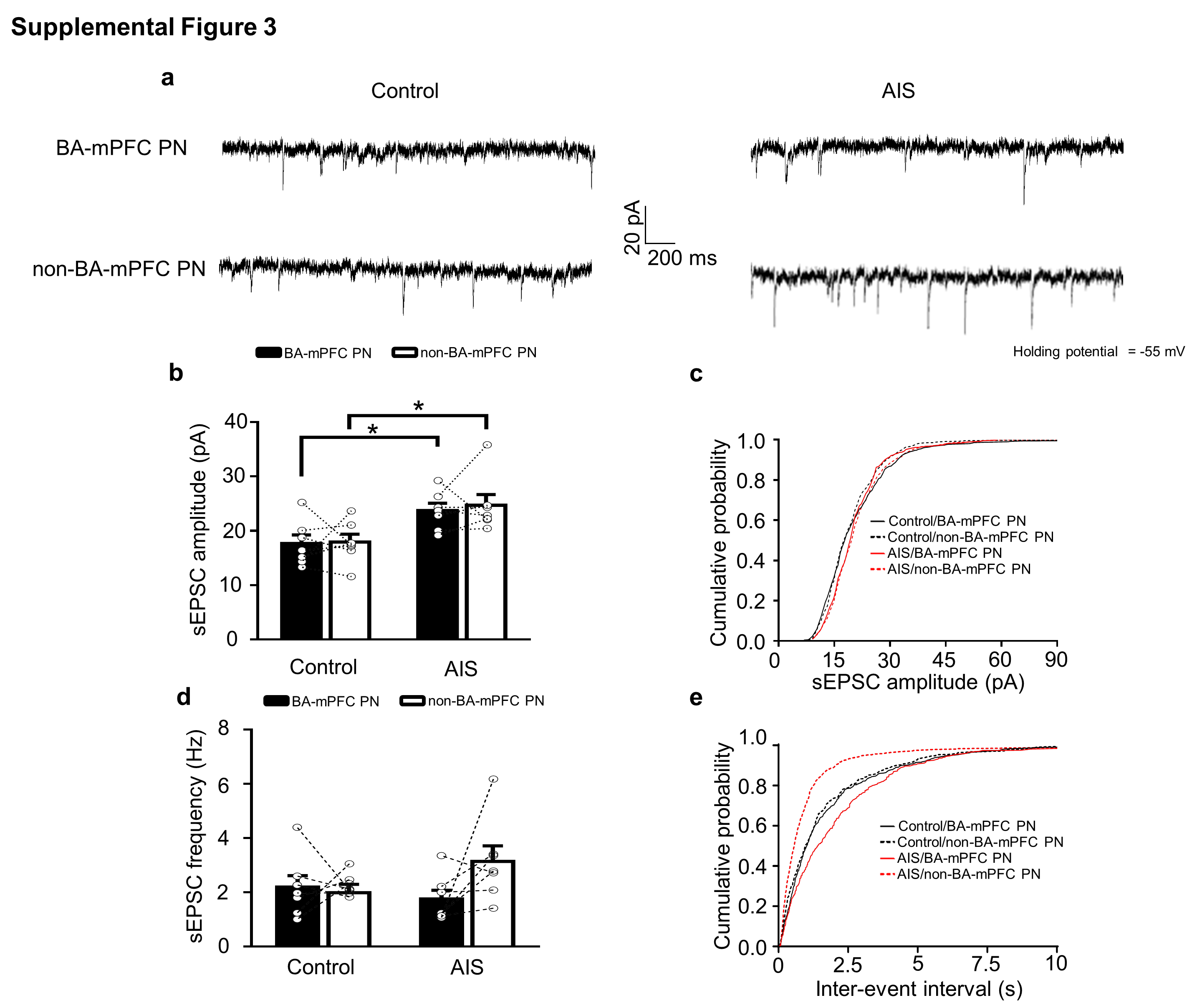

Supplement: Additional file 3: Figure S3. — FSS significantly augments the glutamatergic transmission onto both BA-mPFC and non-BA-mPFC neurons. a Representative traces showing sEPSCs recorded from the BA-mPFC PNs and their neighboring non-BA-mPFC PNs in the control and FSS mice. b Summary data showing the sEPSCs amplitude of the pair-recorded BA-mPFC and non-BA-mPFC PNs (in circle) from the control and AIS mice. Their mean values were shown in column. c Cumulative distribution of the sEPSC amplitude in the two BA neuronal subsets. d Summary data of the sEPSC frequency in both BA neuronal subsets. e Cumulative distribution of the sEPSC frequency in both BA neuronal subsets. *p < 0.05. (TIF 1114 kb) [file 13041_2016_283_MOESM3_ESM.tif]

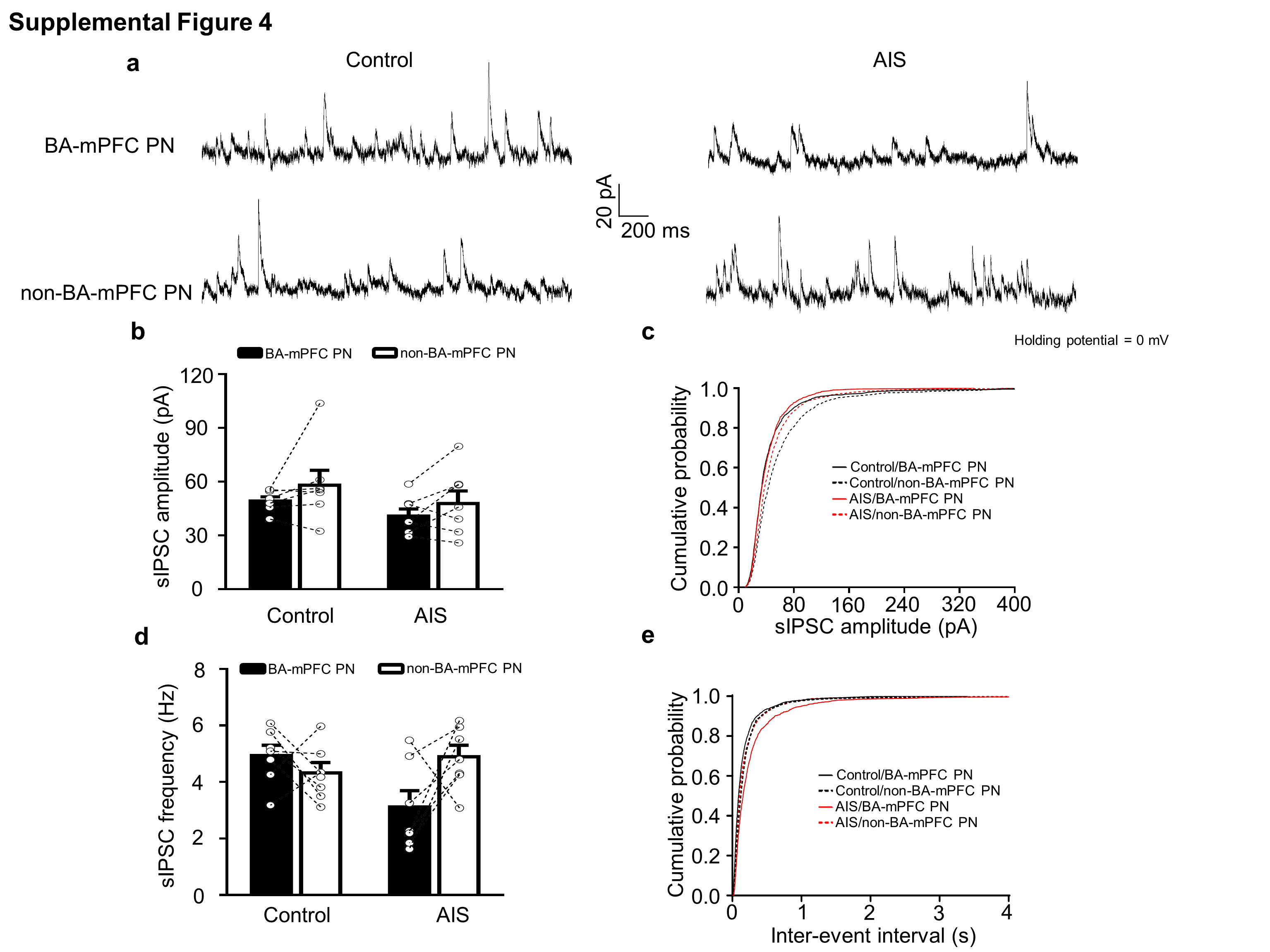

Supplement: Additional file 4: Figure S4. — FSS slightly affects the GABAergic transmission onto both BA-mPFC and non-BA-mPFC neurons. a Representative traces showing sIPSCs recorded from the BA-mPFC PNs and their proximal non-BA-mPFC PNs in control and FSS mice. b Summary data showing the sIPSCs amplitude of the pair-recorded BA-mPFC and non-BA-mPFC PNs (in circle) from the control and AIS mice. Their mean values were shown in column. c Cumulative distribution of the sIPSC amplitude in both BA neuronal subsets. d Summary data of sIPSC frequency in both BA neuron subsets. e Cumulative distribution of the sIPSC frequency in both BA neuron subsets. (TIF 1126 kb) [file 13041_2016_283_MOESM4_ESM.tif]
